# Supplementary figures and images for: A Novel ZnO Nanoparticles Enhanced Surfactant Based Viscoelastic Fluid Systems for Fracturing under High Temperature and High Shear Rate Conditions: Synthesis, Rheometric Analysis, and Fluid Model Derivation
Source: Polymers (Basel). 2022 Sep 26;14(19):4023. doi: 10.3390/polym14194023 (PMC9571908; doi:10.3390/polym14194023)

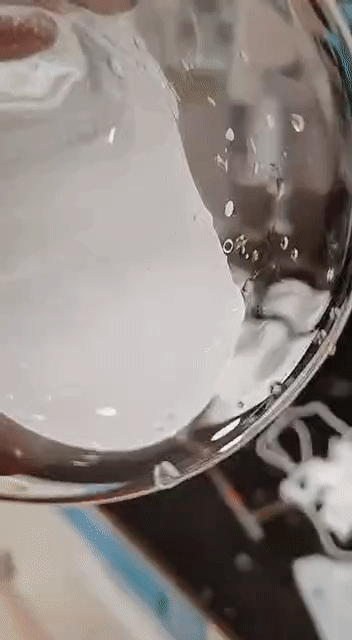

Supplement: Supplementary file 1 [file polymers-14-04023-s001.zip › polymers-1856133-supplementary.gif]
